# Second Opinion in the Italian Organ Procurement Transplantation: The Pathologist Is In

**DOI:** 10.3390/clinpract13030055

**Published:** 2023-04-28

**Authors:** Albino Eccher, Deborah Malvi, Luca Novelli, Claudia Mescoli, Antonietta D’Errico

**Affiliations:** 1 Department of Pathology and Diagnostics and Public Health, Section of Pathology, University Hospital of Verona, 37136 Verona, Italy; 2 Second Opinion, National Transplant Center, 00161 Rome, Italy; 3 Pathology Unit, Department of Specialized, Experimental and Diagnostic Medicine, IRCCS, Azienda Ospedaliero-Universitaria di Bologna, 40138 Bologna, Italy; 4 Institute of Histopathology and Molecular Diagnosis, Careggi University Hospital, 50134 Florence, Italy; 5 Surgical Pathology and Cytopathology Unit, Department of Medicine, University and Hospital Trust of Padua, 35128 Padua, Italy

**Keywords:** second opinion, transplantation, risk assessment

## Abstract

Second opinion consultation is a well-established practice in different clinical settings of diagnostic medicine. However, little is known about second opinion consultation activity in transplantation, and even less is known about it concerning donor assessment. The consultations provided by the second opinion service led to the safer and homogeneous management of donors with a history of malignancy or ongoing neoplasm by transplant centers. Indeed, two of the most important aspects are the reduction of semantic differences in cancer reporting and the standardization of procedures, which are mainly due to the different settings and logistics of different pathology services. This article aims to discuss the role and the future of the second opinion in Italy during organ procurement, highlighting the critical issues and areas for improvement.

## 1. Introduction

The term “second opinion” has been defined in various ways in the literature. A more general definition can be found in the article by Hillen et al.: “Second opinion is when a patient, or a physician, or a “payer” (i.e., a health insurer or a hospital) solicits the assessment of a diagnosis or treatment proposal by a second, independent physician within the same specialty as the physician who gave the “first opinion” [1]. When a second opinion is not sought with the intention of returning it to the initial physician, it is also called “tertiary referral.” Second opinion consultation is a well-established practice in different clinical settings of diagnostic medicine [2]. While the availability of images has simplified access and the protocols of consultations in radiology, with variable implications in different subspecialty areas [3,4,5], this has also become true in pathology, with some studies exploring the issue of discordance and impact on patients’ treatment [6,7,8,9] or legal issues [10,11]. Moreover, the innovation and development of digital pathology have increased opportunities for second opinion consultation when reviewing complex cases and more practically for routine activities or intraoperative frozen sections [12] and on-site diagnostics [13,14]. However, little is known about second opinion consultation activity in transplantation, and even less is known about it in donor assessment. Indeed, in recent decades, the shortage of organs has prompted the use of donors with a prior history of neoplasm or with a neoplastic process discovered at the time of donor evaluation [15]. The greatest risk in transplanting a solid organ from a donor with malignancy is the transmission of the neoplasm to the recipient, with increases in morbidity and mortality. However, this risk has to be balanced against the morbidity and mortality odds of keeping a patient on a waiting list, which has huge costs and a dramatic impact on patients’ quality of life and survival. The overall risk reported in the literature is low but more important for specific types of cancers [16,17,18,19,20,21,22,23].

## 2. History, Role, and Advantages

In 2004, the National Italian Transplant Center decided to create a second opinion consultation service, charged by the Ministry of Health and based on expertise in diagnostic oncology and risk transmission available to the Transplant Coordination Regional Centers. The consultation requests are managed by a pathologist, who is a pivotal figure in the process and who defines the donor’s risk profile, helping centers to evaluate donors’ medical history and to plan clinical, radiological, or pathological investigations. The impact of correct risk assessment is paramount, considering the possible recovery of organs that would have been erroneously discarded or, conversely, the possibly correct discarding of donors with unacceptable risk profiles [24,25,26]. The consultations provided by the second opinion service led to a safer and more homogeneous management of donors by transplant centers. The evaluation of such a donor could cause difficulties in many cases. The opportunity to convey the donors’ data from disparate transplant centers to a unique point represented by the National Second Opinion is paramount for homogenizing procedures for investigating donors for risk assessment. Specific experiences from 2006 to 2015 show that using neoplastic donors is safe since no transmission cases were recorded from this donor pool, expanding the pool of organs for transplant. An overall low transmission rate (0.03%) has demonstrated the efficacy and accuracy of the established protocols of donor assessment. Most transmission cases involved an unrecognized hematologic disease, suggesting exploring improvements to tests to exclude hematologic disease.

Limitations on the recovery of past medical history and intraoperative diagnosis prompted specific educational programs to standardize reports and to improve expertise and reproducibility [15]. Indeed, two of the most important aspects are reducing typology and semantics in cancer reporting and standardizing the procedures, which are mainly due to the different settings and logistics of different pathology services [27]. The role of the National Second Opinion is also in the drafting of the national guidelines to indicate shared procedures and to standardize the preparation methods used in the various pathological anatomy laboratories and, as much as possible, the “forms” for histological reporting of morpho-functional biopsies in cases of neoplasia or suspected neoplasia. As a rule, the second opinion expert confronts and assists the network of colleagues required to evaluate the risk of a donor with neoplasm and guides, with recognized expertise, the entire team of healthcare professionals involved in the potential donor’s management. The ultimate goal of the second opinion is to contribute to the assessment of the tumor risk profile of the potential donor. From a distinctly operational perspective, this is a crucial step that enables operational decisions for all other healthcare professionals involved during donor evaluation and after donor acceptance, with downstream consequences on the organization of intensive care services, operating room availability for organ recovery, and transplant recipients’ information and preparation. At this point, it should be noted that it is always ultimately the responsibility of the clinician managing the potential recipient to evaluate the risk differential between remaining on the waiting list for an indeterminate time and receiving an organ potentially capable of transmitting a neoplasm.

Consultations and answers provided by second opinion experts guarantee the best achievable rapidity in reliable risk assessment, allowing the correct allocation of organs with a collateral positive impact on managing waiting lists, since different recipients in different clinical conditions may or may not receive different organs from donors with different risk profiles. Moreover, the homogeneity of the risk definition coming from a second opinion response assures homogeneity even in recipient management. This benefit adds value in terms of saving time, since many decisions in transplant activity are strictly time-dependent for their outcome and cost-effectiveness, since healthcare systems with limited resources need to manage their different activity networks cost-effectively.

## 3. Critical Issues and Rooms for Improvement

There are several critical areas where the process could be improved:

- The service’s operating methods: Second opinion experts are now available 24 h a day by telephone, which is still a reliable medium in a country such as Italy with geographical and logistic difficulties and differences among regions, providing real-time availability for every center in every region, despite logistical problems. However, transplant pathology aims to become digital [28] by establishing national networks of subspecialist pathologists to support nationwide out-of-hours histopathology for emergency frozen sections and critical decisions [29]. This transition would lead to greater efforts by the entire healthcare system to overcome differences between regions and to create a uniform practice background among centers, with obvious positive consequences for safety throughout the entire process. Furthermore, the introduction of digital pathology has catalyzed the application of artificial intelligence (AI) with the development of novel machine-learning models for tissue interrogation and discovery. Such technological advances offer the potential to improve the ability to classify disease, more accurately quantify morphological alterations, discover correlations with pathogenesis and clinical data, and predict disease outcomes with new prediction models [30]. Regarding the question of when machine-learning algorithms will be ready for use in this setting, we know that the application of AI tools depends on two main factors. The first is the implementation of digital pathology networks. The second is the development of robust and validated AI tools, which should not only replicate experts, but also be directly trained to predict the desired transplant outcome endpoints.
- Standardization: There is often variation in how diagnostic second opinions are conducted, potentially leading to inconsistencies and errors. Social interactions and unrelated conversations often interfere with the process and reduce second opinion quality [31]. Standardizing the process and establishing clear guidelines can help ensure that the second opinion is thorough and accurate. Ensuring that both parties perform their checks of the available data independently can prevent them from potentially following the same reasoning, minimizing the chance of errors [32].
- Data integration: Records may be fragmented or difficult to access, and auto-processing can be an issue. This issue might involve the two people managing a second opinion, where one simply reads the available medical data and the other simply nods in assent. Improving data integration and interoperability can make it easier for specialists to review relevant information and provide an accurate second opinion [33].
- Communication: Effective communication among players is essential for a successful second opinion, with several themes that can contribute to the failure of a second opinion. One theme was deference to authority, which occurs when the individual asked to perform the second opinion is perceived to be below them in the “hierarchy,” sometimes related to their formal title or status. It should be noted that double checks are a form of social redundancy and basically involve one fallible person monitoring the work of another fallible person. When people hear and see what they expect to see, their effectiveness is reduced [34]. Improving communication channels and ensuring that all parties are committed and on the same wavelength can ensure tracking and safety.
- Checklist: Checklists add a cognitive element to oversee tasks or projects and ensure nothing important is forgotten during execution [35]. This way, nothing that might compromise the results is omitted. Additionally, they ensure activities are completed in an orderly, organized manner.
- Transplant outcome tracking: It is vital to track the outcomes of diagnostic second opinions to ensure they effectively improve network management and transplant procedure safety. This tracking can help identify areas for improvement and ensure that the process continues to evolve and improve over time.

## 4. Future Directions

Timing is critical in transplant procurement since organs must be recovered as soon as possible to ensure the best possible outcome for the recipient and to ensure the success of the transplant. Transplant cases are often critical and require immediate consultation with strict turn-around times. The lack of standardized criteria and the scarcity of available expertise add to the dilemma. As a result, the pathologist’s evaluation is often a bottleneck in the process, affecting the entire transplant management. To address this clinical need for the transplant network, implementing a telepathology system is crucial since it enables the network to rapidly obtain a second opinion consultation from a remotely located expert. Implementing digital pathology can be costly since it typically requires the purchase of scanners, computers, and infrastructure for image acquisition, storage, and delivery. In most cases, several factors could result in a return on investment [36,37,38]. Because both radiology and pathology are subspecialties dealing with image-intensive data, some authorities have advocated for joint radiology/pathology programs to manage the large images produced by scanners [39,40]. However, using portable, inexpensive tablets for transplant pathology diagnosis has been validated, given the noninferiority of whole-slide imaging versus light microscopy [41].

Furthermore, digital pathology is important in accelerating healthcare progression, and AI-powered computational pathology can effectively improve diagnostic needs, supporting the quality and safety of the network. As these AI tools are developed, the focus will be on developing ever more accurate models. However, successful translation to the transplant community will also depend upon other system characteristics. The key to unlocking trust will be designing platforms or tools optimized for intuitive human–AI interactions and ensuring that, where judgment is required to resolve ambiguous assessment areas, the tool’s working mode is understandable to the human observer [42]. The goal is to release targeted and “real intelligence” algorithms for organ donor assessment parameters, such as fibrosis, inflammation, and steatosis. There are many advantages to applying AI to organ donor procurement. They include improved accuracy and reproducibility (AI algorithms can analyze images more accurately and quickly than humans), automation (AI can automate tasks such as object detection, image segmentation, and image classification, reducing the need for human intervention and increasing efficiency, especially in organ donor assessments), and improved safety (AI can reduce errors and support live quality control assessment). Furthermore, data integration pipelines could be made available so that centers could have mechanisms for mining data within their center and sharing data with other centers.

## 5. Conclusions

The current scenario in transplant pathology highlights the need to deliver expertise rapidly. Overcoming this issue could lead to a better risk assessment for every donor, with the second opinion service retaining its fundamental value in harmonizing practices and centers. In addition, with the single aim of improving pathologist support to the transplant network, the horizon of the second opinion group is permeated today more than ever by an innovative approach that considers behavioral interactions, social patterns, and technology solutions.

## Data Availability

Not applicable.
